# ADP ribosylation factor–like GTPase 6–interacting protein 5 (Arl6IP5) is an ER membrane-shaping protein that modulates ER-phagy

**DOI:** 10.1016/j.jbc.2025.108493

**Published:** 2025-04-08

**Authors:** Yasunori Yamamoto, Toshiaki Sakisaka

**Affiliations:** Division of Membrane Dynamics, Department of Physiology and Cell Biology, Kobe University School of Medicine, Kobe, Japan

**Keywords:** endoplasmic reticulum (ER), membrane protein, protein domain, membrane structure, autophagy

## Abstract

The endoplasmic reticulum (ER) is the membrane-bound organelle characterized by the reticular network of tubules. It is well established that the ER tubules are shaped by ER membrane proteins containing the conserved reticulon-homology domain (RHD). Membrane shaping by the RHD-containing proteins is also involved in the regulation of ER-phagy, selective autophagy of the ER. However, it remains unclear whether there exists ER membrane-shaping proteins other than the RHD-containing proteins. In this study, we characterize Arl6IP5, an ER membrane protein containing the conserved PRA1 domain, as an ER membrane-shaping protein. Upon overexpression, Arl6IP5 induces the extensive network of the ER tubules and constricts the ER membrane as judged by exclusion of a luminal ER enzyme from the ER tubules. The membrane constriction by Arl6IP5 allows the cells to maintain the tubular ER network in the absence of microtubules. siRNA-mediated knockdown of Arl6IP5 impairs the ER morphology, and the phenotype of the Arl6IP5 knockdown cells is rescued by exogenous expression of Arl6IP1, an RHD-containing protein. Furthermore, exogenous expression of Arl6IP5 rescues the phenotype of Arl6IP1 knockdown cells, and the PRA1 domain is sufficient to rescue it. Upon disruption of the possible short hairpin structures of the PRA1 domain, Arl6IP5 abolishes membrane constriction. The siRNA-mediated knockdown of Arl6IP5 impairs flux of the ER-phagy mediated by FAM134B. These results indicate that Arl6IP5 acts as an ER membrane-shaping protein involved in the regulation of ER-phagy, implying that the PRA1 domain may serve as a general membrane-shaping unit other than the RHD.

The endoplasmic reticulum (ER) is the largest organelle regulating various cellular processes including synthesis and quality control of proteins, lipid biosynthesis, vesicle traffic, calcium storage, autophagy, and immune response (1, 2, 3, 4, 5, 6, 7). The ER is the continuous membrane system composed of two building blocks, ER tubules and ER sheets (8, 9, 10, 11). The ER tubules connect to each other through three-way junctions, thereby generating the reticular network of the ER tubules throughout the cytoplasm (8, 9, 10, 11).

It has been well established that the ER tubules and the edges of ER sheets, both of which are characterized by high curvature membrane, are shaped by two protein families, reticulons and REEPs (9, 10, 12, 13, 14, 15, 16). Reticulons and REEPs are the ER membrane proteins whose primary structures are characterized by the reticulon-homology domain (RHD) (8, 9, 10, 11, 12, 13). While reticulons and REEPs are conserved from yeasts to mammals, mammalian cells also employ Arl6IP1, another RHD-containing protein, to shape the ER tubules (17). The RHD is composed of two long hydrophobic regions flanking a hydrophilic loop of 60 to 70 amino acids (18, 19, 20). Each hydrophobic region does not span the lipid bilayer but adopts a short hairpin configuration, thereby residing in the outer leaflet of the lipid bilayer (9, 13, 14, 18, 21). Newly synthesized RHD-containing proteins are inserted into the outer leaflet of the ER membrane (13, 21, 22) and form the oligomers, which in turn expand the area of the outer leaflet relative to the inner leaflet, thereby generating high curvature membrane such as ER tubules and the edges of ER sheets (9, 12, 13, 14, 18, 21, 22, 23). Indeed, the previous studies have demonstrated that, upon overexpression, RHD-containing proteins such as reticulons and ADP ribosylation factor–like GTPase 6–interacting protein 1 (Arl6IP1) induces the extensive network of the peripheral ER tubules and strongly constricts the ER membrane, leading to exclusion of luminal proteins from the peripheral ER tubules (13, 17, 18, 24). These activities of reticulons and Arl6IP1 are abolished by disruption of the short hairpin configurations of RHD (17, 18).

ER homeostasis is maintained by ER-phagy, selective autophagy of the ER (25, 26, 27). Evidence is accumulating that ER-phagy employs RHD-containing proteins for autophagosome formation (28, 29, 30, 31, 32, 33). FAM134B and Rtn3L, both of which are the RHD-containing proteins localized at the ER membrane, act as ER-phagy receptors (28, 30, 32). FAM134B and Rtn3L recruit the autophagy machinery to the ER membrane by binding to LC3 on the phagophore membrane and then cause scission of the ER membrane through the RHD, leading to autophagosome formation (28, 30, 32). Arl6IP1 forms the heteromeric clusters with FAM134B on the ER membrane, leading to enhancement of the ER-phagy (33).

ADP ribosylation factor–like GTPase 6–interacting protein 5 (Arl6IP5), also known as PRAF3, GTRAP3-18, JWA, and addicsin, is the ER membrane protein and has been shown to be involved in the regulation of various biological processes. A well-characterized function of Arl6IP5 is the regulation of glutamate transport in neurons (34, 35, 36). Arl6IP5 binds to EAAC1, a sodium-dependent glutamate transporter, and inhibits the ER export of EAAC1 (34, 35, 36). The inhibition of EAAC1 by Arl6IP5 is suggested to be involved in the regulation of glutathione synthesis in neurons, because EAAC1 transports glutamate and cysteine, both of which are the constituent amino acids of glutathione (37, 38, 39). Arl6IP5 is also involved in the regulation of bone formation. Arl6IP5 modulates the ER stress response and expression of RANKL, an osteoclastogenic cytokine, leading to regulation of osteoblast differentiation and osteoclastogenesis (40). Arl6IP5 is implicated in the progression of Parkinson's disease (PD) (41). The expression level of Arl6IP5 decreases in the brain of the PD patients, which leads to accumulation of α-synuclein aggregates through downregulation of autophagy (41).

The primary structure of Arl6IP5 is characterized by the PRA1 domain encompassing four transmembrane domains (TMDs) (42). Interestingly, the first TMD is very close to the second TMD, because the distance between the TMDs is only five amino acids. Therefore, we cannot rule out the possibility that the first and second TMDs might act as if they were a single long hydrophobic region. Similarly, the third and fourth TMDs are also very close to each other. Therefore, these configurations of the TMDs raise the possibility that the PRA1 domain of Arl6IP5 might be composed of two long hydrophobic regions. The PRA1 domain is conserved from yeasts to mammals (42, 43, 44) and defined as a region that has the significant sequence similarity to prenylated Rab acceptor protein 1 (PRA1) (also known as RABAC1 and PRAF1). PRA1, Arl6IP5, and PRAF2, another membrane protein having the PRA1 domain, comprise the PRA1 domain family (PRAF) in mammals (42, 43, 44). It has been demonstrated that PRA1 binds to prenylated Rab GTPases and regulates cycling of Rab GTPases between membrane-bound and soluble forms by functioning as a GDI-displacement factor (45). However, it remains unclear whether the other family members, Arl6IP5 and PRAF2, function as GDI-displacement factor. While the PRA1 domain does not show significant sequence similarity to the RHD, a recent review article has suggested the possibility that PRAF members might be involved in shaping membrane in a similar manner to reticulons by adopting two short hairpin configurations in the PRA1 domain (46). The earlier study and BioGRID, the database for protein and genetic interactions, have shown interactions between Arl6IP5 and the ER membrane-shaping proteins such as Arl6IP1, reticulons, REEP5, and atlastin-2 (47, 48, 49, 50). Given that the ER membrane-shaping proteins have a tendency to interact with each other (13, 17, 51), these interactions of Arl6IP5 seem to support the idea that Arl6IP5 might be involved in shaping the ER membrane. However, since this idea has never been experimentally demonstrated, it remains unknown whether Arl6IP5 indeed acts as an ER membrane-shaping protein or not.

In this study, we characterize Arl6IP5 in the context of a potential ER membrane-shaping protein. We show that overexpression of Arl6IP5 induces peripheral ER tubules and allows cells to retain the peripheral tubular network even in the absence of microtubules, which are reminiscent of the overexpression of Arl6IP1 or reticulons. siRNA-mediated knockdown of Arl6IP5 impaired the tubular ER network. The tubular ER network impaired by Arl6IP5 knockdown is rescued by exogenous expression of Arl6IP1. Furthermore, exogenous expression of Arl6IP5 rescues the tubular ER network impaired by Arl6IP1 knockdown, and the PRA1 domain is sufficient to rescue it. The membrane-shaping activity of Arl6IP5 relies on the possible short hairpin structures of the PRA1 domain. Arl6IP5 knockdown decreases flux of the ER-phagy mediated by FAM134B. These results indicate that Arl6IP5 acts as an ER membrane-shaping protein involved in the regulation of ER-phagy.

## Results

### Overexpression of Arl6IP5 induces the extensive network of peripheral ER tubules

**Figure 1:**
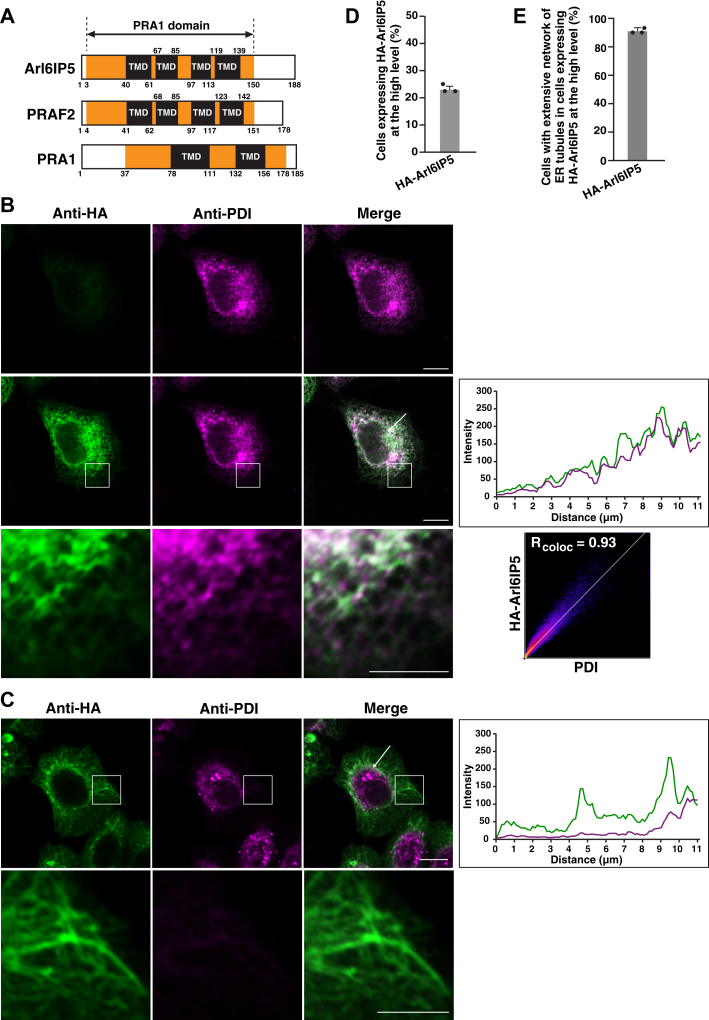
**Overexpression of Arl6IP5 induces the extensive network of the peripheral ER tubules.***A*, the domain structures of human PRAF proteins. Arl6IP5, PRAF2, and PRA1 are composed of the PRA1 domain harboring transmembrane domains (TMDs). The position of the PRA1 domain is based on SMART (Simple Modular Architecture Research Tool) (https://smart.embl.de). The positions of TMDs are predicted by Phobius (https://phobius.sbc.su.se/). The numbers indicate the positions of the amino acid residues. Arl6IP5 and PRAF2 are predicted to have four TMDs. PRA1 is predicted to have two TMDs, presumably because Phobius recognizes two tandem TMDs as a single, long TMD. *B*, localization of Arl6IP5 at the ER. HeLa cells were transfected with HA-Arl6IP5, followed by immunostaining with the anti-HA mAb and the anti-PDI mAb. The images of the cell expressing HA-Arl6IP5 at the low level are shown. The *upper panel*s are the images taken with the same setting of the confocal microscope (such as laser power, and gain and offset parameters) as those of the cells expressing HA-Arl6IP5 at the high level as shown in (*C*). The *middle panels* are the images taken by adjusting the microscope settings to detect ER localization of HA-Arl6IP5. Bars represent 10 μm. The boxed area is enlarged and shown in the *bottom panels*. Bar represents 5 μm. The *rightmost* graph in the *middle panels* shows fluorescence intensity profiles for HA-Arl6IP5 and PDI along the *white arrow*. The *rightmost* graph in the *bottom panels* shows the cytofluorogram and Rcoloc indicates Pearson's correlation coefficient. The images and results shown are representative of three independent experiments. *C*, induction of the extensive network of the peripheral ER tubules and exclusion of PDI from the peripheral ER tubules by overexpression of Arl6IP5. HeLa cells were subjected to transfection followed by immunostaining as in (*B*). The images of the cell expressing HA-Arl6IP5 at the high level are shown. The images are taken with the same setting of the confocal microscope as the images in the *upper panels* in (*B*), highlighting the difference in extent of expression of HA-Arl6IP5. Bar represents 10 μm. The boxed area is enlarged and shown in the *bottom panels*. Bar represents 5 μm. The *rightmost* graph in the *upper panels* shows fluorescence intensity profiles for HA-Arl6IP5 and PDI along the *white arrow*. The images and results shown are representative of three independent experiments. *D*, ratio of the cells expressing HA-Arl6IP5 at the high level. Sixty transfected cells were randomly chosen and the number of the cells expressing HA-Arl6IP5 at the high level was counted. The mean value and SD of three independent experiments is shown. Each dot represents the result of a single experiment. *E*, ratio of the cells having the extensive network of ER tubules induced by the overexpression of HA-Arl6IP5. Thirty cells expressing HA-Arl6IP5 at the high level were randomly chosen and the number of the cells having the extensive network of ER tubules, as judged by the presence of unbranched, long ER tubules, was counted. The mean value and SD of three independent experiments is shown. Each dot represents the result of a single experiment.

Arl6IP5 is the ER membrane protein whose primary structure is characterized by the PRA1 domain harboring four transmembrane domains (Fig. 1*A*). In this study, we set out to examine whether Arl6IP5 had the ability to shape the ER membrane. It is well known that, upon overexpression, RHD-containing proteins such as Arl6IP1 and reticulons induces the extensive network of the peripheral ER tubules, leading to the formation of unbranched, long ER tubules, and exclude luminal proteins such as protein disulfide isomerase (PDI), a luminal ER enzyme, from the peripheral ER tubules by strongly constricting the ER membrane (13, 17, 18, 24). The previous studies used HeLa cells or COS-7 cells to demonstrate the *in vitro* activities of RHD-containing proteins (13, 17, 18, 24). We therefore examined whether overexpression of Arl6IP5 had the similar effects to that of Arl6IP1 in HeLa cells. HA-tagged Arl6IP5 (HA-Arl6IP5) or HA-tagged Arl6IP1 (HA-Arl6IP1) was transfected into HeLa cells, followed by immunostaining with the anti-HA mAb and the anti-PDI mAb. Consistent with the previous study (17), HA-Arl6IP1 localized at the ER membrane as judged by PDI staining in the cells expressing HA-Arl6IP1 at low levels (Fig. S1*A*). On the other hand, it was hard to see the ER localization of HA-Arl6IP1, when the images of the cells expressing HA-Arl6IP1 at the low levels were taken with the same setting of the confocal microscope (such as laser power, and gain and offset parameters) as those of the cells expressing HA-Arl6IP1 at the high levels (Fig. S1*A*, upper panels). These results highlight the difference in the extent of expression. About 28% of the transfected cells showed the high level expression of HA-Arl6IP1 (Fig. S1*C*), and the high level expression of HA-Arl6IP1 frequently induced the extensive network of the peripheral ER tubules as characterized by the presence of unbranched, long ER tubules and excluded PDI from the peripheral ER tubules (Fig. S1*B*). To quantify the effect of the high level expression of HA-Arl6IP1 on ER morphology, cells having the extensive network of ER tubules were judged by the presence of unbranched, long ER tubules, and the number of the cells was counted. About 83% of cells expressing the high level of HA-Arl6IP1 showed the extensive network of ER tubules as judged by the presence of unbranched, long ER tubules (Fig. S1*D*). The cells with unbranched, long ER tubules showed exclusion of PDI from the peripheral ER tubules. Consistent with the previous study (17), these results indicate that, upon overexpression, Arl6IP1 induced extensive network of ER tubules and constricted the ER membrane. In agreement with the previous finding that Arl6IP5 is the ER membrane protein (36, 52), HA-Arl6IP5 localized at the ER membrane in the cells expressing HA-Arl6IP5 at low levels (Fig. 1*B*). It was hard to see the ER localization of HA-Arl6IP5, when the images of the cells expressing HA-Arl6IP5 at the low levels were taken with the same setting of the confocal microscope as those of the cells expressing HA-Arl6IP5 at the high levels (Fig. 1*B*, upper panels). These results highlight the difference in the extent of expression. Importantly, the high level expression of HA-Arl6IP5 induced the extensive network of the peripheral ER tubules as characterized by the presence of unbranched, long ER tubules and excluded PDI from the peripheral ER tubules (Fig. 1*C*). About 23% of the transfected cells expressed the high level of HA-Arl6IP5 (Fig. 1*D*), and about 90% of cells expressing the high level of HA-Arl6IP5 showed the extensive network of ER tubules as judged by the presence of unbranched, long ER tubules (Fig. 1*E*). The cells with unbranched, long ER tubules showed exclusion of PDI from the peripheral ER tubules. These effects of HA-Arl6IP5 are similar to those of HA-Arl6IP1, suggesting that Arl6IP5 has the activity to induce the extensive network of the peripheral ER tubules and constrict the ER membrane.

### Arl6IP5 has the ability to shape the peripheral ER tubules

It is well known that the ER membrane is physically associated with microtubules, and the microtubules are required to maintain the ER tubules *in vivo* (21). However, overexpression of the RHD-containing proteins such as Arl6IP1 and reticulons has been shown to allow cells to maintain the ER tubules in the absence of microtubules, because reticulons or Arl6IP1 strongly constrict the ER membrane into tubules (17, 21). We here employed this assay to further assess whether Arl6IP5 has the activity to constrict the ER membrane into tubules.

**Figure 2:**
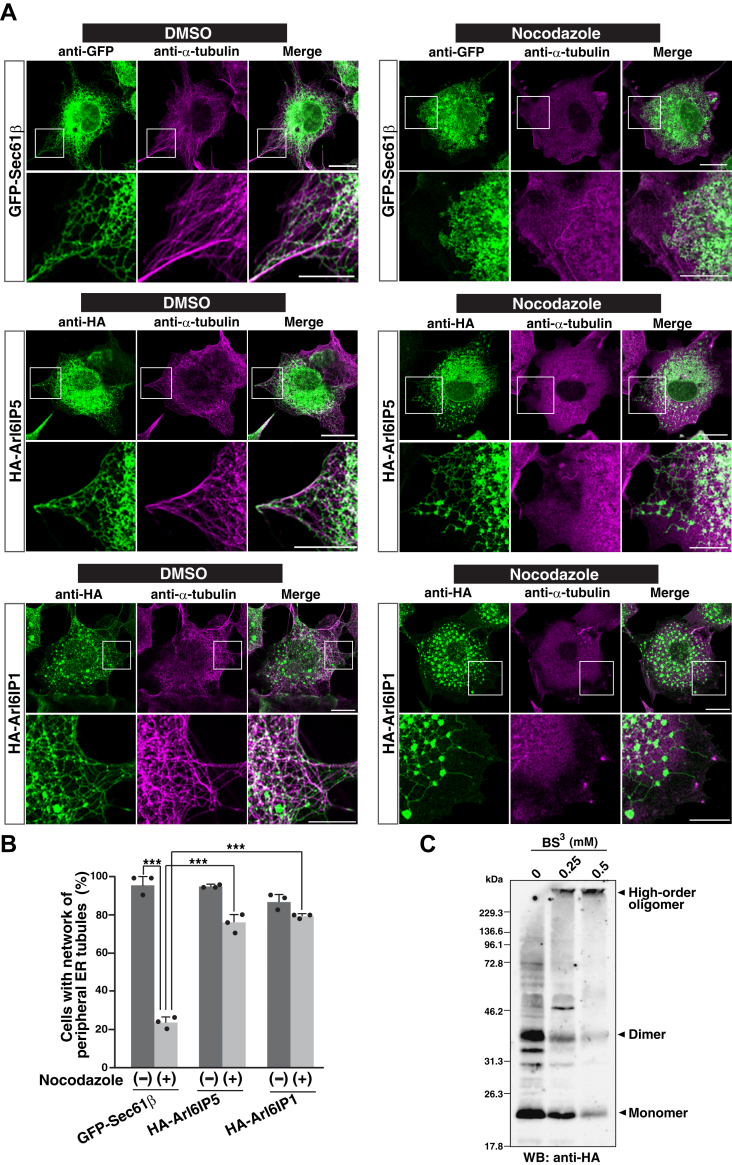
**Arl6IP5 has the ability to shape the peripheral ER tubules**. *A*, overexpression of Arl6IP5 allows cells to maintain the ER tubules without microtubules. COS-7 cells were transfected with HA-Arl6IP5, HA-Arl6IP1, or GFP-Sec61β and cultured in the presence or absence of nocodazole, followed by immunostaining with the anti-HA mAb or the anti-GFP mAb and the anti-α-tubulin mAb. The *upper panels* are the images of the GFP-Sec61β–transfected cells. The *middle panels* are the images of HA-Arl6IP5–transfected cells. The *bottom panels* are the images of HA-Arl6IP1–transfected cells. Bars represents 20 μm. The boxed area is enlarged and shown beneath each image. Bars represents 10 μm. The results shown are representative of three independent experiments. *B*, quantification of the effect of overexpression of Arl6IP5 on the peripheral ER tubules. In (*A*), total 50 transfected cells were randomly chosen in each experiment, and the number of cells having the network of peripheral ER tubules was counted. Error bars represent SD of three independent experiments. Each dot represents the result of a single experiment. Statistical analysis was performed using one-way ANOVA (F = 258, *p* = 8.88 × 10^−12^) followed by Bonferroni's *post hoc* test (*p* = 2.30 × 10^−5^ (GFP-Sec61β in the presence of nocodazole *versus* GFP-Sec61β in the absence of nocodazole), *p* = 5.45 × 10^−5^ (HA-Arl6IP5 in the presence of nocodazole *versus* GFP-Sec61β in the presence of nocodazole), *p* = 7.65 × 10^−6^ (HA-Arl6IP1 in the presence of nocodazole *versus* GFP-Sec61β in the presence of nocodazole)). ∗∗∗*p* < 0.001. *C*, Arl6IP5 forms high-order oligomers on the ER membrane. COS-7 cells transfected with HA-Arl6IP5 were disrupted by hypotonic buffer, and the membrane fraction was isolated by ultracentrifugation. The membrane fraction was then incubated with the indicated concentrations of BS^3^, a chemical cross-linker, for 30 min, followed by immunoblotting with the anti-HA mAb. The result shown is representative of two independent experiments.

Since the previous studies used COS-7 cells to demonstrate the microtubule-independent shaping of ER membrane (17, 21), we transfected HA-Arl6IP5 or GFP-tagged Sec61β (GFP-Sec61β), a control ER membrane protein, into COS-7 cells. The cells were then cultured in the presence of nocodazole to allow the cells to depolymerize microtubules, followed by immunostaining with the anti-HA mAb or the anti-GFP mAb and the anti-α-tubulin mAb. The cells transfected with GFP-Sec61β showed the reticular network of ER tubules in the peripheral region in the absence of nocodazole (Fig. 2*A*, upper panels). Immunostaining for α-tubulin confirmed that nocodazole disrupted microtubules, and disruption of microtubules by nocodazole completely abolished the peripheral ER tubules and their network in most of the cells transfected with GFP-Sec61β as shown in Figure 2*A*, upper panels. To quantify the effect of nocodazole on ER morphology, the number of cells having the network of peripheral ER tubules was counted. Indeed, about 95% of the cells transfected with GFP-Sec61β had the network of peripheral ER tubules in the absence of nocodazole, whereas only 23% of those had the network of peripheral ER tubules in the presence of nocodazole (Fig. 2*B*). Importantly, the cells transfected with HA-Arl6IP5 frequently showed the network of peripheral ER tubules even in the presence of nocodazole as shown in Figure 2*A*, middle panels. In agreement, the number of cells having the network of peripheral ER tubules in the presence of nocodazole was significantly increased by the transfection of HA-Arl6IP5 relative to that of GFP-Sec61β (Fig. 2*B*). In addition, it was confirmed that transfection of HA-Arl6IP1 has similar effect to that of HA-Arl6IP5 (Fig. 2*A*, bottom panels, and Fig. 2*B*). These results indicate that overexpression of Arl6IP5 allows cells to maintain the ER tubules in the absence of microtubules, similarly to the overexpression of Arl6IP1.

To further confirm the ability of Arl6IP5 to shape the ER tubules, we examined whether Arl6IP5 formed the oligomer on the ER membrane, because ER membrane-shaping proteins such as reticulons and Arl6IP1 are well known to form oligomers in order to shape the ER membrane (17, 21). COS-7 cells transfected with HA-Arl6IP5 were disrupted by hypotonic buffer, and the membrane fraction was isolated by ultracentrifugation. The membrane fraction was then incubated with various concentrations of BS^3^, a chemical cross-linker, followed by immunoblotting with the anti-HA mAb. Consistent with the earlier finding that Arl6IP5 forms SDS-resistant dimers (53), not only the 20 kDa band of an HA-Arl6IP5 monomer but also the 40 kDa band indicative of an SDS-resistant dimer were detected without BS^3^ (Fig. 2*C*). Importantly, BS^3^ reduced both the monomer and dimer bands and instead increased an immunoreactive band more than 230 kDa, which indicated the high-order oligomer, in a concentration-dependent manner (Fig. 2*C*). These results indicate that Arl6IP5 forms the oligomer on the ER membrane. Collectively, these results indicate that Arl6IP5 has the ability to shape the peripheral ER tubules.

### Arl6IP5 is required for formation of the tubular ER network

**Figure 3:**
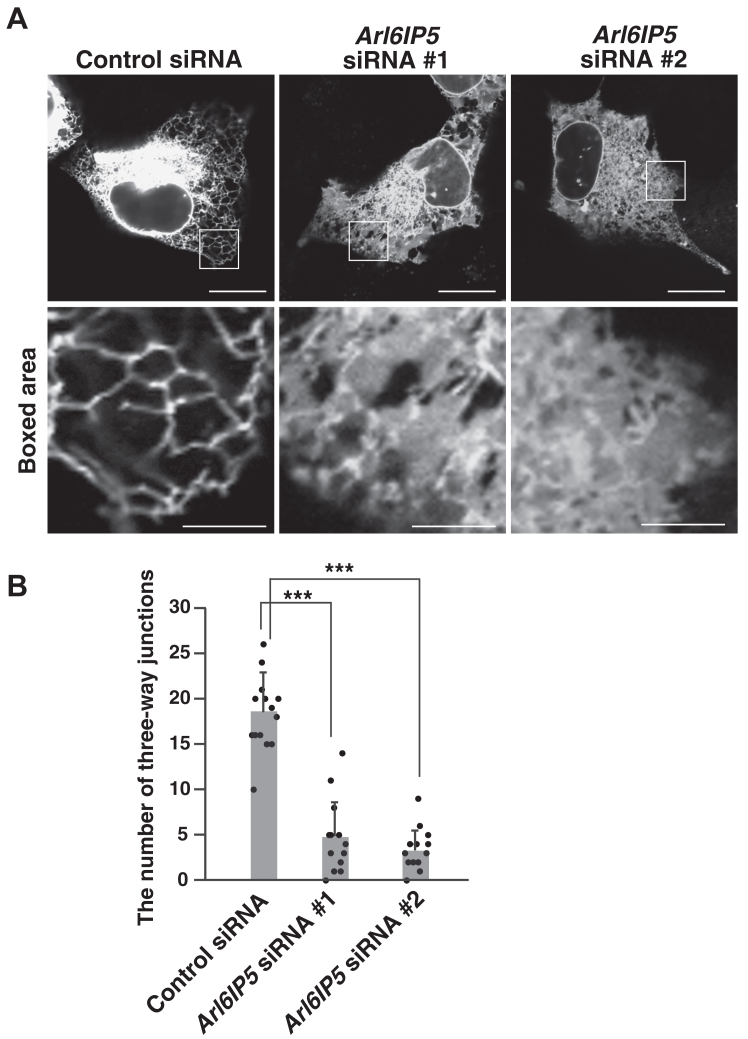
**Arl6IP5 is required for formation of the tubular ER network**. *A*, Arl6IP5 knockdown impairs the tubular ER network. U2OS cells were transfected with siRNAs targeting *Arl6IP5* or the control siRNA and cultured for 2 days. The cells were transfected with GFP-Sec61β, and the GFP fluorescence was analyzed in the live cells. The results shown are representative of three independent experiments. Bars represent 20 μm. The boxed area is enlarged and shown beneath each image. Bars represent 5 μm. *B*, quantification of the effect of Arl6IP5 knockdown on the peripheral network of ER tubules. In (*A*), total 15 transfected cells were randomly chosen, and the number of three-way junctions in an area of 10 μm × 10 μm in the peripheral region was counted. Each dot represents the number of three-way junctions of a single cell. Bar graphs represent mean values. Error bars represent SDs. Statistical analysis was performed using one-way ANOVA (F = 90.0, *p* = 6.52 × 10^−6^) followed by Bonferroni's *post hoc* test (*p* = 1.87 × 10^−10^ (*Arl6IP5* siRNA #1 *versus* control siRNA), *p* = 2.56 × 10^−13^ (*Arl6IP5* siRNA #2 *versus* control siRNA)). ∗∗∗*p* < 0.001.

We examined whether Arl6IP5 was required for formation of the tubular ER network. U2OS cells are often used to examine the effects of depletion of endogenous proteins on the ER morphology (33, 54, 55, 56, 57), because the size of U2OS cells is larger than other human cell lines such as HeLa and HEK293 cells. The large cell size allows us to readily assess the peripheral network of ER tubules. In addition, U2OS cells express endogenous Arl6IP5. We therefore employed U2OS cells to examine the effects of Arl6IP5 knockdown on formation of the peripheral network of ER tubules. U2OS cells were transfected with siRNAs targeting *Arl6IP5* to allow knockdown of endogenous Arl6IP5. The knockdown of Arl6IP5 was confirmed by quantitative RT-PCR (Fig. S2). The Arl6IP5 knockdown cells or the cells transfected with the control siRNA were then transfected with GFP-Sec61β to visualize the ER, followed by the detection of GFP fluorescence in live cells. The cells transfected with the control siRNA showed the reticular signal of GFP-Sec61β in the peripheral region, indicating formation of the peripheral tubular ER network (Fig. 3*A*). By contrast, the Arl6IP5 knockdown cells frequently abolished the reticular signal of GFP-Sec61β and increased the GFP-Sec61β–positive area in the cytoplasm (Fig. 3*A*). A tubule to sheet transition is the expected phenotype caused by the reduction of ER membrane-shaping proteins and has been observed with the RHD-containing proteins (23, 58, 59). Therefore, the increased area of GFP-Sec61β will be explained by the tubule to sheet transition. To quantify the effect of Arl6IP5 knockdown, the number of three-way junctions was counted in a given area of the peripheral region. The average number of three-way junctions was significantly decreased in the cells transfected with *Arl6IP5* siRNA #1 or *Arl6IP5* siRNA #2 relative to the cells transfected with the control siRNA (Fig. 3*B*). The decrease in the number of three-way junctions is in good agreement with disruption of the tubular ER network by Arl6IP5 knockdown as shown in Figure 3*A* and indicate that Arl6IP5 is required for formation of the tubular ER network.

### Arl6IP5 and Arl6IP1 are redundant in shaping the ER membrane

**Figure 4:**
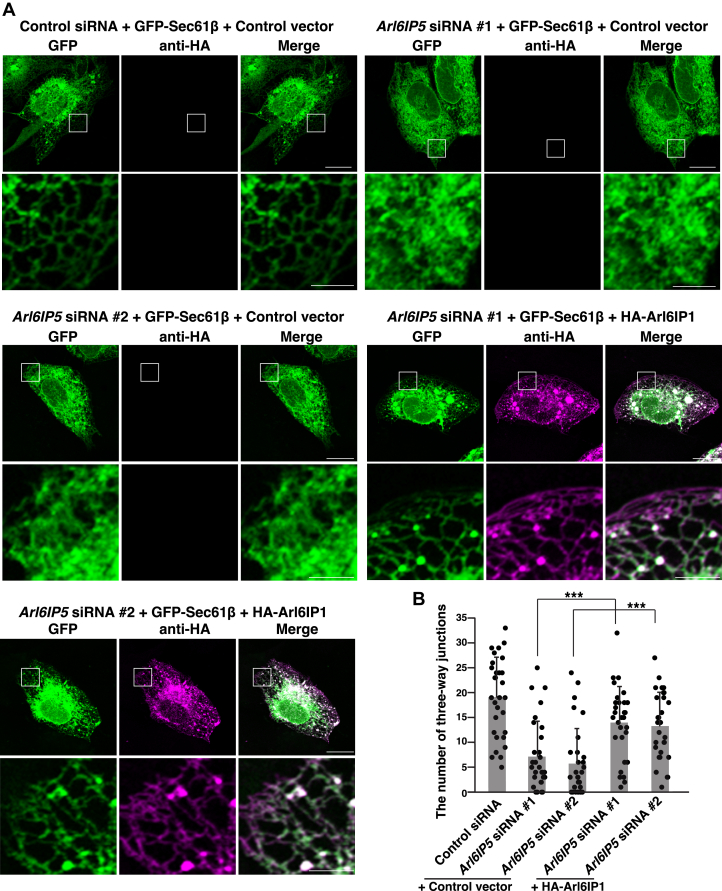
**The impaired ER morphology in Arl6IP5 knockdown cells is rescued by exogenous expression of Arl6IP1**. *A*, Arl6IP1 can rescue the phenotype of the Arl6IP5 knockdown cells. U2OS cells were transfected with siRNAs targeting *Arl6IP5* and cultured for 2 days to allow knockdown of endogenous Arl6IP5. HA-Arl6IP1 or the control vector was transfected into the Arl6IP5 knockdown cells along with GFP-Sec61β, followed by immunostaining with the anti-HA mAb. GFP fluorescence was used for detection of GFP-Sec61β. Bars represent 20 μm. The *boxed area* is enlarged and shown beneath each image. Bars represent 5 μm. The results shown are representative of three independent experiments. *B*, quantification of the effect of exogenous expression of Arl6IP1 on the peripheral network of ER tubules. In (*A*), total 30 transfected cells were randomly chosen, and the number of three-way junctions in an area of 10 μm × 10 μm in the peripheral region was counted. Each dot represents the number of three-way junctions of a single cell. Bar graphs represent mean values. Error bars represent SDs. Statistical analysis was performed using one-way ANOVA (F = 17.2, *p* = 1.44 × 10^−11^) followed by Bonferroni's *post hoc* test (*p* = 4.01 × 10^−4^ (*Arl6IP5* siRNA #1 + HA-Arl6IP1 *versus Arl6IP5* siRNA #1 + control vector), *p* = 7.70 × 10^−5^ (*Arl6IP5* siRNA #2 + HA-Arl6IP1 *versus Arl6IP5* siRNA #2 + control vector)). ∗∗∗*p* < 0.001.

We reasoned that, if Arl6IP5 acted as an ER membrane-shaping protein like RHD-containing proteins, the ER morphology impaired by Arl6IP5 knockdown would be rescued by exogenous expression of the RHD-containing proteins. To examine this reasoning, HA-Arl6IP1 or the control vector was transfected into the Arl6IP5 knockdown cells along with GFP-Sec61β. The cells were then fixed, followed by immunostaining with the anti-HA mAb. GFP fluorescence was used for the detection of GFP-Sec61β. Consistent with the results of the live cells as shown in Figure 3, the Arl6IP5 knockdown cells transfected with the control vector showed the impaired ER structure as characterized by the abolishment of the reticular signal of GFP-Sec61β (Fig. 4*A*) and decreased the average number of three-way junctions relative to the cells transfected with the control siRNA and the control vector (Fig. 4*B*). By contrast, the Arl6IP5 knockdown cells transfected with HA-Arl6IP1 frequently showed the reticular signal of GFP-Sec61β indicative of the tubular ER network (Fig. 4*A*). In agreement, the Arl6IP5 knockdown cells transfected with HA-Arl6IP1 significantly increased the average number of three-way junctions relative to the Arl6IP5 knockdown cells transfected with the control vector (Fig. 4*B*). These results indicate that the exogenous expression of Arl6IP1 rescued the reticular ER structure impaired by Arl6IP5 knockdown.

**Figure 5:**
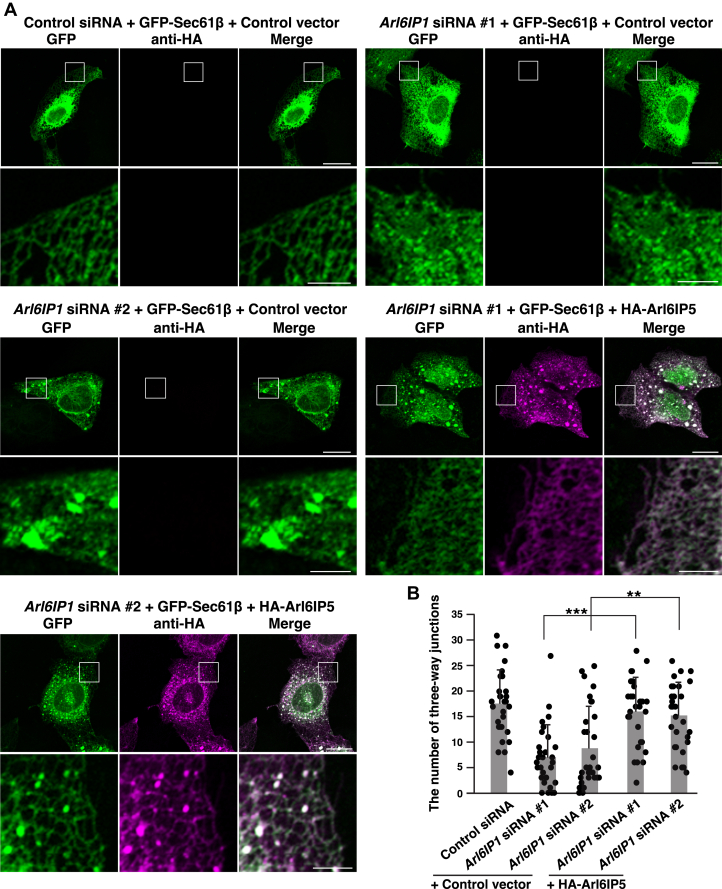
**The impaired ER morphology in Arl6IP1 knockdown cells is rescued by exogenous expression of Arl6IP5**. *A*, Arl6IP5 can rescue the phenotype of the Arl6IP1 knockdown cells. U2OS cells were transfected with siRNAs targeting *Arl6IP1* and cultured for 2 days to allow knockdown of endogenous Arl6IP1. HA-Arl6IP5 or the control vector was transfected into the Arl6IP1 knockdown cells along with GFP-Sec61β, followed by immunostaining with the anti-HA mAb. GFP fluorescence was used for the detection of GFP-Sec61β. Bars represent 20 μm. The boxed area is enlarged and shown beneath each image. Bars represent 5 μm. The results shown are representative of three independent experiments. *B*, quantification of the effect of exogenous expression of Arl6IP5 on the peripheral network of ER tubules. In (*A*), total 30 transfected cells were randomly chosen and the number of three-way junctions in an area of 10 μm × 10 μm in the peripheral region was counted. Each dot represents the number of three-way junctions of a single cell. Bar graphs represent mean values. Error bars represent SDs. Statistical analysis was performed using one-way ANOVA (F = 14.7, *p* = 3.97 × 10^−10^) followed by Bonferroni's *post hoc* test (*p* = 4.81 × 10^−7^ (*Arl6IP1* siRNA #1 + HA-Arl6IP5 *versus Arl6IP1* siRNA #1 + control vector), *p* = 1.10 × 10^−3^ (*Arl6IP1* siRNA #2 + HA-Arl6IP5 *versus Arl6IP1* siRNA #2 + control vector)). ∗∗∗*p* < 0.001. ∗∗*p* < 0.01.

We next examined whether exogenous expression of Arl6IP5 rescued the phenotype of Arl6IP1 knockdown cells. U2OS cells were transfected with siRNAs targeting *Arl6IP1* to allow knockdown of endogenous Arl6IP1. The knockdown of Arl6IP1 was confirmed by quantitative RT-PCR (Fig. S3). HA-Arl6IP5 or the control vector was transfected into the Arl6IP1 knockdown cells along with GFP-Sec61β, and the cells were analyzed in the same manner as the Arl6IP5 knockdown cells in Figure 4. While the cells transfected with the control siRNA showed the reticular signal of GFP-Sec61β in the peripheral region, Arl6IP1 knockdown cells transfected with the control vector frequently abolished the reticular signal and increased the GFP-Sec61β–positive area in the cytoplasm (Fig. 5*A*). The Arl6IP1 knockdown cells transfected with the control vector decreased the average number of three-way junctions relative to the cells transfected with the control siRNA and the control vector (Fig. 5*B*). These results indicate that, consistent with the previous studies (33, 58), the Arl6IP1 knockdown caused impairment of the reticular network of the ER tubules presumably through the tubule to sheet transition. By contrast, the Arl6IP1 knockdown cells transfected with HA-Arl6IP5 frequently showed the reticular signal of GFP-Sec61β indicative of the tubular ER network (Fig. 5*A*). In agreement, the Arl6IP1 knockdown cells transfected with HA-Arl6IP5 significantly increased the average number of three-way junctions relative to the Arl6IP1 knockdown cells transfected with the control vector (Fig. 5*B*). These results indicate that the exogenous expression of Arl6IP5 rescued the reticular ER structure impaired by Arl6IP1 knockdown. Collectively, these results suggest that Arl6IP5 and Arl6IP1 are redundant in shaping the ER membrane and further support that Arl6IP5 acts as an ER membrane-shaping protein.

### The PRA1 domain is important for Arl6IP5 to shape the ER membrane

**Figure 6:**
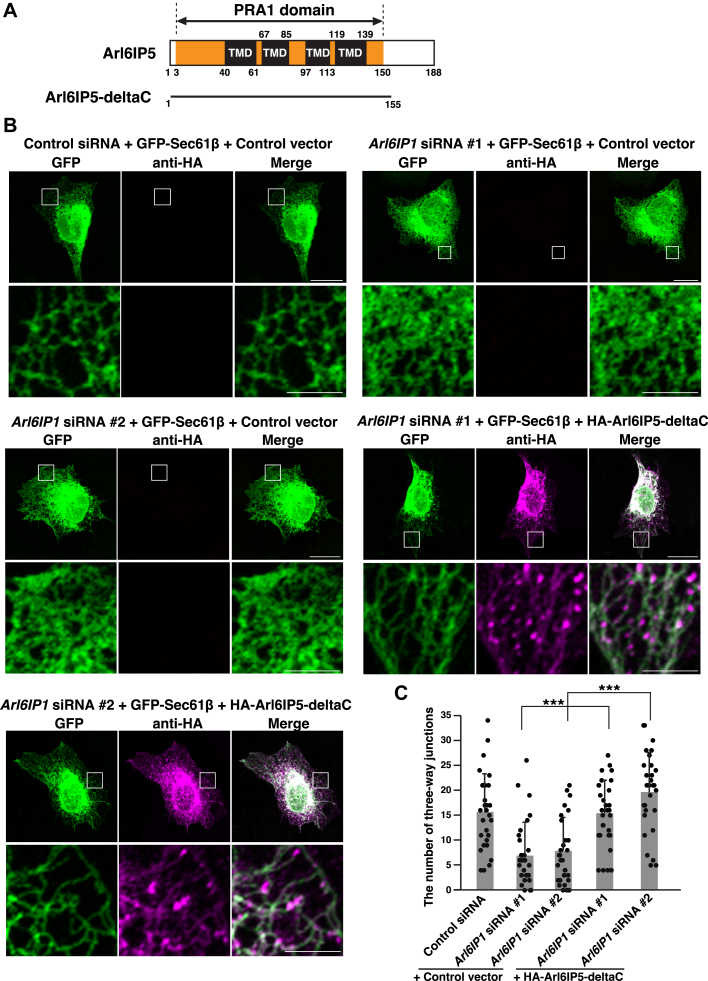
**The PRA1 domain is sufficient to rescue the phenotype of Arl6IP1 knockdown cells.***A*, schematic representation of the C-terminal deletion mutant of Arl6IP5 encompassing the PRA1 domain only; *B*, Arl6IP5-deltaC can rescue the phenotype of the Arl6IP1 knockdown cells. U2OS cells were transfected with siRNAs targeting *Arl6IP1* and cultured for 2 days to allow knockdown of endogenous Arl6IP1. HA-Arl6IP5-deltaC or the control vector was transfected into the Arl6IP1 knockdown cells along with GFP-Sec61β, followed by immunostaining with the anti-HA mAb. GFP fluorescence was used for the detection of GFP-Sec61β. Bars represent 20 μm. The boxed area is enlarged and shown beneath each image. Bars represent 5 μm. The results shown are representative of three independent experiments. *C*, quantification of the effect of exogenous expression of Arl6IP5-deltaC on the peripheral network of ER tubules. In (*B*), total 30 transfected cells were randomly chosen, and the number of three-way junctions in an area of 10 μm × 10 μm in the peripheral region was counted. Each dot represents the number of three-way junctions of a single cell. Bar graphs represent mean values. Error bars represent SDs. Statistical analysis was performed using one-way ANOVA (F = 18.1, *p* = 24.33 × 10^−12^) followed by Bonferroni's *post hoc* test (*p* = 7.58 × 10^−6^ (*Arl6IP1* siRNA #1 + HA-Arl6IP5-deltaC *versus Arl6IP1* siRNA #1 + control vector), *p* = 3.43 × 10^−8^ (*Arl6IP1* siRNA #2 + HA-Arl6IP5-deltaC *versus Arl6IP1* siRNA #2 + control vector)). ∗∗∗*p* < 0.001.

We sought to examine whether the PRA1 domain of Arl6IP5 was involved in membrane shaping. To this end, we generated a deletion mutant of Arl6IP5 encompassing the PRA1 domain only (Arl6IP5-deltaC) (Fig. 6*A*) and examined whether Arl6IP5-deltaC was sufficient to rescue the phenotype of the Arl6IP1 knockdown cells. HA-tagged Arl6IP5-deltaC (HA-Arl6IP5-deltaC) or the control vector was transfected into the Arl6IP1 knockdown cells along with GFP-Sec61β, and the cells were analyzed in the same manner as the Arl6IP5 knockdown cells in Figure 4. While the Arl6IP1 knockdown cells transfected with the control vector caused impairment of the peripheral network of ER tubules, the Arl6IP1 knockdown cells transfected with HA-Arl6IP5-deltaC maintained the peripheral network of ER tubules (Fig. 6*B*). In agreement, while the Arl6IP1 knockdown cells transfected with the control vector decreased the average number of three-way junctions relative to the cells transfected with the control siRNA and the control vector, the Arl6IP1 knockdown cells transfected with HA-Arl6IP5-deltaC significantly increased the average number of three-way junctions relative to the Arl6IP1 knockdown cells transfected with the control vector (Fig. 6*C*). These results indicate that the PRA1 domain was sufficient to rescue the phenotype of Arl6IP1 knockdown cells.

**Figure 7:**
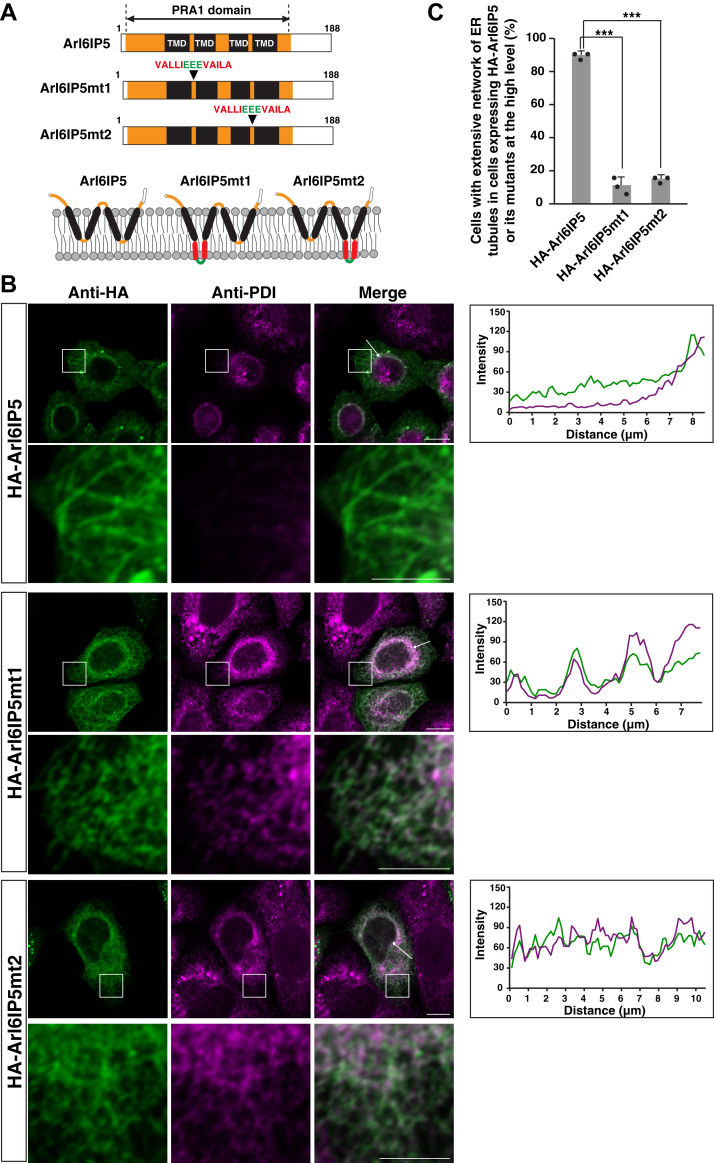
**The possible short hairpin structures of the PRA1 domain are required for the membrane-shaping activity of Arl6IP5**. *A*, schematic representation of the Arl6IP5 mutants disrupting the possible short hairpin structures of the PRA1 domain. The extra amino acids composed of VALLIEEEVAILA are inserted into the PRA1 domain so that Arl6IP5mt1 and Arl6IP5mt2 can have the artificial hydrophobic stretches in the TMDs. The hydrophobic amino acids are indicated in *red*. The hydrophilic amino acids are indicated in *green*. The *bottom panel* depicts the possible short hairpin structures of Arl6IP5 as recently suggested (46) and its disruption in Arl6IP5mt1 and Arl6IP5mt2. *B*, overexpression of Arl6IP5mt1 and Arl6IP5mt2 does not induce the extensive network of the ER tubules and exclude PDI from the peripheral ER tubules. HeLa cells were transfected with HA-Arl6IP5, HA-Arl6IP5mt1, or HA-Arl6IP5mt2, followed by immunostaining with the anti-HA mAb and the anti-PDI mAb. The images of the cells expressing HA-Arl6IP5, HA-Arl6IP5mt1, or HA-Arl6IP5mt2, at the high level are shown. The all images are taken with the same setting of the confocal microscope, demonstrating that the extent of expression of HA-Arl6IP5, HA-Arl6IP5mt1, and HA-Arl6IP5mt2 were similar to each other. Bars represent 10 μm. The boxed area is enlarged and shown beneath each panel. Bars represent 5 μm. The *rightmost* graphs show fluorescence intensity profiles for HA-Arl6IP5, HA-Arl6IP5mt1, or HA-Arl6IP5mt2, and PDI along the *white* arrow. The images and results shown are representative of three independent experiments. *C*, ratio of the cells having the extensive network of ER tubules induced by overexpression of HA-Arl6IP5, HA-Arl6IP5mt1, and HA-Arl6IP5mt2. Thirty cells expressing HA-Arl6IP5, HA-Arl6IP5mt1, or HA-Arl6IP5mt2, at the high level were randomly chosen and the number of the cells having the extensive network of ER tubules, as judged by the presence of unbranched, long ER tubules, was counted. The mean value and SD of three independent experiments is shown. Each dot represents the result of a single experiment. Statistical analysis was performed using one-way ANOVA (F = 515.1, *p* = 1.94 × 10^−7^) followed by Bonferroni's *post hoc* test (*p* = 1.58 × 10^−5^ (HA-Arl6IP5mt1 *versus* HA-Arl6IP5), *p* = 1.26 × 10^−6^ (HA-Arl6IP5mt2 *versus* HA-Arl6IP5)). ∗∗∗*p* < 0.001.

The short hairpin structures of RHD are prerequisite for the membrane-shaping activity of the RHD-containing proteins. Indeed, the previous studies demonstrated that the mutations disrupting the short hairpin structures in RHD abolished the membrane-shaping activity of reticulons and Arl6IP1 (17, 18). The possibility that the PRA1 domain would adopt short hairpin structures similar to RHD has been recently suggested (46). To further validate that the PRA1 domain has the membrane-shaping activity similar to RHD, we generated the Arl6IP5 mutants disrupting the possible short hairpin structures of the PRA1 domain in the same manner as RHD in the previous studies (Fig. 7*A*). These mutants (Arl6IP5mt1 and Arl6IP5mt2) have the artificial hydrophobic stretches in the TMDs of the PRA1 domain, thereby allowing the PRA1 domain to span the ER membrane (Fig. 7*A*). HA-tagged Arl6IP5mt1 (HA-Arl6IP5mt1), HA-tagged Arl6IP5mt2 (HA-Arl6IP5mt2), or HA-Arl6IP5 was transfected into HeLa cells and subjected to immunostaining. Consistent with the results in Figure 1, overexpression of HA-Arl6IP5 induced the extensive network of the peripheral ER tubules as characterized by the presence of unbranched, long ER tubules and excluded PDI from the peripheral ER tubules (Fig. 7*B*). By contrast, overexpression of HA-Arl6IP5mt1 and HA-Arl6IP5mt2 had no effect on the ER network and PDI staining (Fig. 7*B*). In agreement, the number of cells having the extensive network of ER tubules as judged by the presence of unbranched, long ER tubules was significantly lower in the overexpression of HA-Arl6IP5mt1 and HA-Arl6IP5mt2 than overexpression of HA-Arl6IP5 (Fig. 7*C*). These results demonstrate that the possible short hairpin structures of the PRA1 domain are prerequisite for the membrane-shaping activity of Arl6IP5. Collectively, these results indicate the PRA1 domain is important for Arl6IP5 to shape the ER membrane and further support that the PRA1 domain would act as a membrane-shaping unit similar to RHD.

### Arl6IP5 is involved in the regulation of FAM134B-mediated ER-phagy

It has been demonstrated that Arl6IP1 binds to FAM134B, an ER-phagy receptor, and thereby facilitates FAM134B-mediated deformation of the ER membrane, leading to the enhancement of ER-phagy (33). We demonstrate here that Arl6IP5 and Arl6IP1 have redundant roles in shaping the ER membrane as shown in Figures 4 and 5. In addition, the recent proteomic studies have identified Arl6IP5 as a component of the FAM134B complex (32, 33). These results prompted us to examine whether Arl6IP5 is involved in the regulation of ER-phagy. We first assessed the interaction between Arl6IP5 and FAM134B. Since HEK293 cells are widely used to examine protein–protein interaction *in vitro* due to high transfection efficiency, FLAG-tagged Arl6IP5 (FLAG-Arl6IP5) and HA-tagged FAM134B (HA-FAM134B) were transfected into HEK293 cells, followed by immunoprecipitation with the FLAG mAb. HA-FAM134B was significantly coimmunoprecipitated with FLAG-Arl6IP5, indicating that, in agreement with the earlier finding that Arl6IP5 is the component of the FAM134B complex (32, 33), Arl6IP5 bound to FAM134B (Fig. S4*A*). On the other hand, although we carried out immunoprecipitation of endogenous FAM134B from HEK293 lysates with the anti-FAM134B pAb, we could not detect coimmunoprecipitation of endogenous Arl6IP5 (Fig. S4*B*). These conflicting results imply that binding of Arl6IP5 to FAM134B might be weak or transient *in vivo*.

**Figure 8:**
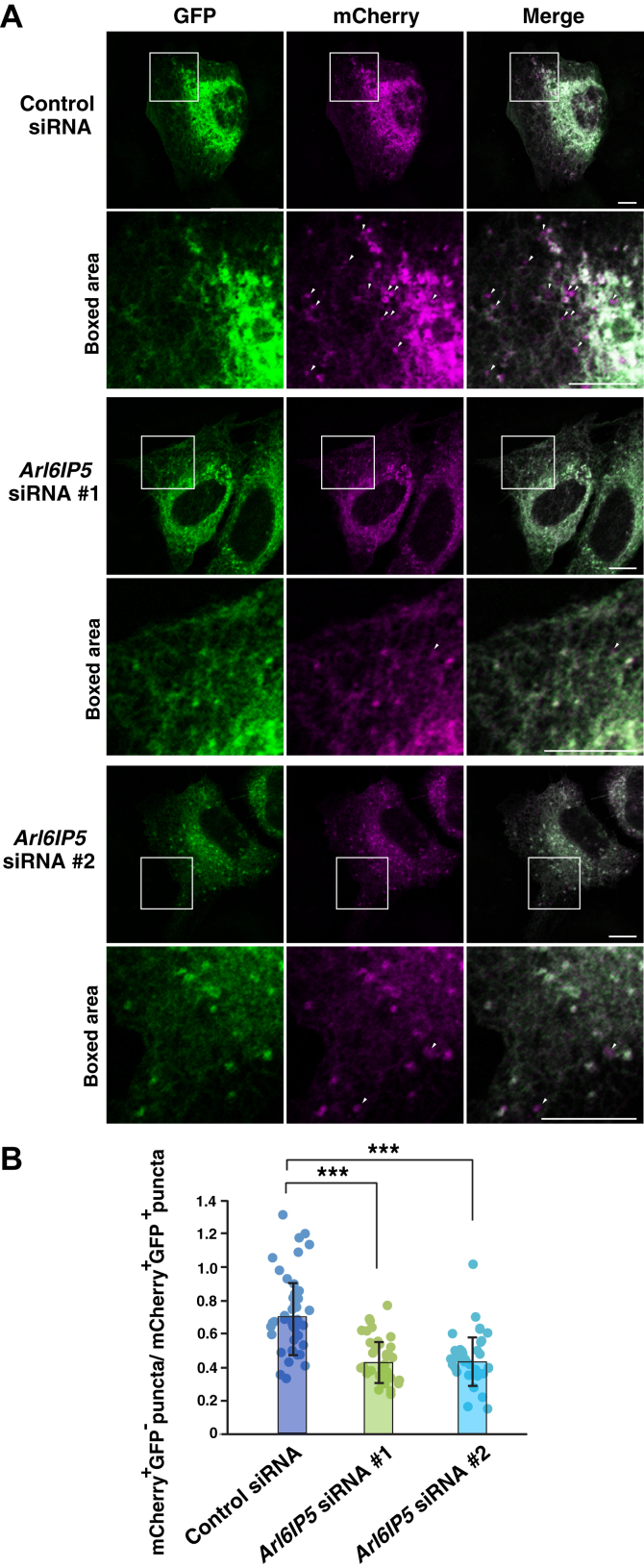
**Arl6IP5 is involved in the regulation of FAM134B-mediated ER-phagy**. *A*, Arl6IP5 siRNA knockdown affects the flux of FAM134B-mediated ER-phagy. U2OS cells were transfected with siRNAs targeting *Arl6IP5* or the control siRNA and cultured for 3 days, followed by transfection with mCherry-GFP-FAM134B. Six hours after transfection of mCherry-GFP-FAM134B, the cells were fixed and mCherry and GFP fluorescence were observed. Bars represent 10 μm. The boxed area is enlarged and shown beneath each image. Arrowheads indicate representative mCherry^+^GFP^-^ puncta. Bars represent 10 μm. *B*, quantification of the effect of Arl6IP5 knockdown on the flux of FAM134B-mediated ER-phagy. In (*A*), cells expressing moderate levels of mCherry-GFP-FAM134B were randomly chosen, and the number of mCherry^+^GFP^-^ puncta and mCherry^+^GFP^+^ puncta was counted. The ratio between mCherry^+^GFP^-^ puncta and mCherry^+^GFP^+^ puncta was then calculated and plotted as a dot. The number of cells analyzed were as follows: 42 (control siRNA), 36 (*Arl6IP5* siRNA #1), 34 (*Arl6IP5* siRNA #2). Bar graphs represent mean values. Error bars represent SDs. Statistical analysis was performed using one-way ANOVA (F = 29.7, *p* = 5.12 × 10^−11^) followed by Bonferroni's *post hoc* test (*p* = 1.34 × 10^−8^ (*Arl6IP5* siRNA #1 *versus* control siRNA), *p* = 1.42 × 10^−7^ (*Arl6IP5* siRNA #2 *versus* control siRNA)). ∗∗∗*p* < 0.001.

We next examined whether Arl6IP5 was involved in the regulation of ER-phagy. To this end, we employed mCherry-EGFP tagged FAM134B (mCherry-EGFP-FAM134B), a well-established reporter to assess the flux of ER-phagy (28, 32, 33, 60, 61). Since mCherry, but not EGFP, is resistant to the acidic environment of lysosomes, the mCherry-EGFP-FAM134B reporter allows us to detect autophagosomes as puncta positive for both mCherry and EGFP and autolysosomes as puncta positive for mCherry only, respectively. Since U2OS cells are often used to demonstrate the effects of depletion of endogenous proteins on the ER-phagy (28, 30, 32, 33, 60), mCherry-EGFP-FAM134B was transfected into Arl6IP5 knockdown cells or control siRNA-transfected cells and fixed 6 hours after transfection to avoid overexpression of mCherry-EGFP-FAM134B, followed by observation of the mCherry and GFP fluorescence. While mCherry-EGFP-FAM134B mostly showed ER localization, mCherry-EGFP-FAM134B puncta indicative of autophagosomes or autolysosomes were also detected (Fig. 8*A*). Compared with the control siRNA-transfected cells, the Arl6IP5 knockdown cells significantly reduced the ratio of mCherry^+^EGFP^−^ puncta to mCherry^+^EGFP^+^ puncta (*i.e.* ratio of autolysosomes to autophagosomes) (Fig. 8*B*). These results indicate that Arl6IP5 is involved in the regulation of FAM134B-mediated ER-phagy.

## Discussion

In this study, we demonstrated that Arl6IP5 acts as an ER membrane-shaping protein for formation of the ER tubules. It is widely accepted that the ER tubules are shaped by RHD-containing proteins such as reticulons, REEPs, and Arl6IP1 (9, 10, 12, 13, 14, 15, 16, 17). The membrane-shaping activity of Arl6IP5 is very similar to that of Arl6IP1, an RHD-containing protein as shown in Figures 1 and 2. Besides, Arl6IP5 and Arl6IP1 are functionally redundant in shaping the ER membrane as shown in Figures 4 and 5. However, Arl6IP5 does not have the RHD. The TMDs of Arl6IP5 are characterized by the PRA1 domain conserved from yeasts to mammals and show the sequence similarity to those of PRA1 and PRAF2. The membrane-shaping activity of Arl6IP5 relies on the PRA1 domain as shown in Figures 6 and 7. Collectively, these results raise an attractive possibility that the PRA1 domain-containing proteins including Arl6IP5, PRA1, and PRAF2 might constitute a membrane-shaping protein family other than the reticulon family, as recently suggested (46). PRA1 is the membrane protein localizing at Golgi and endosomes and suggested to regulate vesicle formation from Golgi (52, 62, 63). Given that depletion of PRA1 does not affect Golgi morphology as demonstrated by the earlier study (64), the possible membrane-shaping activity of PRA1 might be involved in deformation of the Golgi membrane required for vesicle formation. PRAF2 localizes at the ER membrane (42, 44) and is suggested to regulate membrane trafficking of the cell surface receptors (44, 65). However, there is no study investigating the involvement of PRAF2 in formation of the ER morphology so far. Therefore, further studies will be required to examine whether the PRA1 domain generally acts as a general membrane-shaping unit other than the RHD.

Arl6IP5 is involved in the regulation of FAM134B-mediated ER-phagy as shown in Figure 8. While FAM134B is the ER-phagy receptor, FAM134B itself is an RHD-containing protein (26, 27, 28). FAM134B constricts the ER membrane through the RHD during ER-phagy, leading to membrane scission required for autophagosome formation (26, 27, 28). It has been recently reported that the membrane constriction by Arl6IP1 assists FAM134B-mediated ER-phagy (33). Arl6IP1 forms the heteromeric complex with FAM134B on the ER membrane and thereby facilitates the membrane scission for autophagosome formation, resulting in the enhancement of FAM134B-mediated ER-phagy (33). Given that the functional redundancy between Arl6IP5 and Arl6IP1 and *in vitro* binding of Arl6IP5 to FAM134B as shown in Figures 4, 5, and S4*A*, it seems to be plausible that Arl6IP5 would facilitate the FAM134B-mediated ER-phagy in the similar manner to Arl6IP1. On the other hand, although Arl6IP5 has been shown to be the component of the FAM134B complex (32, 33), we could not detect coimmunoprecipitation of endogenous Arl6IP5 and endogenous FAM134B as shown in Fig. S4*B*. These results seem to imply that binding of Arl6IP5 to FAM134B might be weaker or more transient *in vivo* than that of Arl6IP1. Therefore, we cannot also rule out the possibility that Arl6IP5 might regulate the FAM134B-mediated ER-phagy in a different manner from Arl6IP1. The recent studies have demonstrated that monoubiquitination of the RHD-containing proteins regulate ER-phagy (32, 33). Both Arl6IP1 and FAM134B are monoubiquitinated by AMFR, an ER-localized E3 ligase, which in turn promotes formation of the heteromeric complex, leading to the enhancement of autophagosome formation (32, 33). It remains unknown whether the membrane-shaping activity of Arl6IP5 is subject to regulation by monoubiquitination. On the other hand, the previous study has shown that Arl6IP5 is ubiquitinated by RNF185, another ER-localized E3 ubiquitin ligase, leading to proteasomal degradation of Arl6IP5 (66). Therefore, unlike ubiquitination of Arl6IP1, ubiquitination of Arl6IP5 might be involved in downregulation of the FAM134B-mediated ER-phagy.

The siRNA-mediated knockdown of Arl6IP5 impaired the ER morphology as shown in Figure 3, indicating that Arl6IP5 plays a predominant role in shaping the ER membrane. The previous study has shown that genetic ablation of Arl6IP5 impairs differentiation of osteoblasts by inducing ER stress (40), underscoring the importance of Arl6IP5 in bone formation. The ER stress response is associated with the morphological change of the ER (11). Therefore, there is a possibility that the morphological change of the ER caused by Arl6IP5 depletion might excessively activate the ER stress response, leading to impaired differentiation of osteoblasts. On the other hand, evidence is accumulating that ER-phagy ameliorates ER stress (67, 68). Therefore, another possibility is that induction of ER stress in the Arl6IP5 KO mice might be attributed in part to reduction of the FAM134B-mediated ER-phagy. However, it remains unknown whether the ER morphology and ER-phagy are impaired in osteoblasts of the Arl6IP5 KO mice. Further studies will be required to understand involvement of the membrane-shaping activity of Arl6IP5 in the differentiation of osteoblasts. It has been shown that oxidative stress induces the expression of Arl6IP5 (69). Given that autophagy is upregulated under oxidative stress conditions (70), induction of Arl6IP5 might be involved in boosting FAM134B-mediated ER-phagy in response to oxidative stress. It is known that pathogenic mutations of the genes encoding Arl6IP1 and FAM134B cause hereditary spastic paraplegias and hereditary sensory and autonomic neuropathy type II, respectively (71, 72). We show here that Arl6IP5 and Arl6IP1 play functionally redundant roles in shaping the ER membrane and that Arl6IP5 regulates FAM134B-mediated ER-phagy, raising the possibility that Arl6IP5 might be also involved in these neurodegenerative disorders. Further studies will be required to address these concerns. In summary, we show here that Arl6IP5 acts as an ER membrane-shaping protein involved in the regulation of ER-phagy.

## Experimental procedures

### Plasmids

The complementary DNAs (cDNAs) encoding human Arl6IP5 and human FAM134B were subcloned into pCMV vectors with N-terminal HA or FLAG tags. The cDNA encoding the deletion mutant of Arl6IP5 (Arl6IP5-deltaC) (1–155 aa) was amplified by PCR using full-length human Arl6IP5 as a template and subcloned into the pCMV vector with the N-terminal HA tag. For Arl6IP5mt1 and Arl6IP5mt2, the extra amino acids composed of VALLIEEEVAILA were inserted between proline at 63 aa residue and phenylalanine at 64 aa residue in the PRA1 domain of human Arl6IP5 and between methionine at 115 aa residue and phenylalanine at 116 aa residue in the PRA1 domain of human Arl6IP5, respectively, by site-directed mutagenesis. pCMV-HA-Arl6IP1 and pEGFP-C1-Sec61β have been generated in our previous study (17). For mCherry-GFP-FAM134B, the cDNA encoding human FAM134B was subcloned into pEGFP-C1 (CLONTECH). The cDNA encoding GFP-FAM134B was amplified by PCR using pEGFP-C1-FAM134B as a template and subcloned into the pCMV5-mCherry vector.

### Antibodies

A mouse anti-PDI monoclonal Ab (mAb), a rat anti-HA mAb, a mouse anti-HA mAb, a rat anti-GFP mAb, and a rabbit anti-FAM134B polyclonal Ab (pAb) were purchased from Abcam, Roche, BioLegend, Nacalai Tesque, and Proteintech, respectively. A mouse anti-FLAG mAb, a rabbit anti-FLAG pAb, a mouse anti-α-tubulin mAb, and a rabbit anti-Arl6IP5 pAb were purchased from SIGMA.

### Immunofluorescence microscopy

HeLa cells, U2OS cells, and COS-7 cells were maintained in Dulbecco's modified Eagle's medium supplemented with 10% fetal bovine serum, and appropriate plasmids were transfected with Effectene (Qiagen). In some instances, cells were cultured in the presence of 1 μM nocodazole for 30 min to allow depolymerization of the microtubules. The cells were fixed with 4% paraformaldehyde, followed by permeabilization with 0.2% Triton X-100. After blocking with PBS supplemented with 1% bovine serum albumin, the samples were incubated with primary Abs, followed by incubation with Alexa Fluor–conjugated secondary Abs (Invitrogen). After being washed with PBS, they were embedded and viewed using a confocal imaging system (ZEISS, LSM 510 Meta, or LSM 710). Image J was used to obtain the fluorescence intensity profiles, the cytofluorogram, and Pearson's correlation coefficient.

It is well known that overexpression of the RHD-containing proteins in HeLa cells causes induction of the extensive network of ER tubules, leading to the formation of unbranched, long ER tubules. Therefore, to quantify the effects of overexpression of Arl6IP5 and Arl6IP1 on the ER morphology, the cells inducing the extensive network of ER tubules were judged by the presence of the unbranched, long ER tubules, and the number of the cells was counted. The cells with unbranched, long ER tubules also showed exclusion of PDI from the peripheral ER tubules, which was another phenotype caused by the overexpression of RHD-containing proteins. While COS-7 cells show the reticular network of ER tubules in the peripheral region, disruption of microtubules by nocodazole completely abolishes the ER tubules in most of the cells. Therefore, to quantify the effects of overexpression of Arl6IP5 and Arl6IP1 on the microtubule-independent formation of ER tubules, the number of cells having the network of peripheral ER tubules was counted in the presence or absence of nocodazole.

### Chemical cross-linking experiments

The crude membrane fractions of COS-7 cells were isolated and chemically cross-linked as reported previously with a slight modification (17, 21). Briefly, HA-Arl6IP5 was transfected into COS-7 cells using PEI in a 10-cm dish. The day after transfection, the cells were harvested and incubated with hypotonic buffer (10 mM Hepes–KOH pH 7.4, 10 mM potassium acetate, 1.5 mM magnesium acetate, 2 mM PMSF) for 10 min and then passed through a 25-gauge syringe ten times. The nuclei and cell debris were removed from the lysates by centrifugation at 3000*g* for 5 min, and the supernatant was centrifuged at 100,000*g* for 10 min to pellet the membrane fraction. After being washed with HKM buffer (25 mM Hepes–KOH pH 7.4, 150 mM potassium acetate, 2.5 mM magnesium acetate, 2 mM PMSF), the membrane fractions were resuspended in 100 μl of HKM buffer. Twenty microliters of the membrane fractions was incubated with various concentrations of BS^3^ (Bis(sulfosuccinimidyl) suberate) (PIERCE) for 30 min at room temperature to allow the chemical cross-linking reaction to proceed. After the reaction was terminated by the addition of 10 mM Tris–HCl pH 7.5, the samples were subjected to SDS-PAGE followed by immunoblotting with the anti-HA mAb.

The blot was developed by chemiluminescence (Nacalai Tesque, Chemi-Lumi One Super) and the blot image was captured by LAS-4000 (Cytiva).

### siRNA knockdown

U2OS cells were transfected with 10 μM of the silencer-select siRNAs targeting human *Arl6IP5* (Thermo Fisher Scientific, Cat. No. 4427037, ID s20689 for siRNA#1, ID s20690 for siRNA#2), the silencer-select siRNAs targeting human *Arl6IP1* (Thermo Fisher Scientific, Cat. No. 4427037, ID s23274 for siRNA#1, ID s23275 for siRNA#2), or the control siRNA (Thermo Fisher Scientific, Cat. No. AM4613) using Lipofectamine RNAi MAX (Invitrogen) in accordance with the manufacturer's manual. The siRNA sequences used are as follows: 5′-aaauggaaggaauagguuutt-3′ (*Arl6IP5* siRNA#1), 5′-ggaauagguuugaagaggatt-3′ (*Arl6IP5* siRNA#2), 5′-ggacuaaaccaacauggaatt-3′ (*Arl6IP1* siRNA#1), 5′-aaaccuaagauguacuucatt-3′ (*Arl6IP1* siRNA#2).

Two days after transfection with the *Arl6IP5* siRNAs, the cells were transfected with GFP-Sec61β and further cultured for 24 h to allow the expression of GFP-Sec61β. The live cells were directly viewed using a confocal imaging system (ZEISS, LSM700). Areas of 10 μm × 10 μm in the cell periphery were randomly selected, and the number of three-way junctions was counted. The number of three-way junctions was used to quantify the effects of Arl6IP5 knockdown on the ER morphology.

In some instances, the cells were transfected with appropriate combinations of GFP-Sec61β, the control vector, HA-Arl6IP1, HA-Arl6IP5, and HA-Arl6IP5-deltaC 2 days after transfection with the *Arl6IP5* siRNAs or the *Arl6IP1* siRNAs and further cultured for 24 h to allow expression of the transfected genes. After being fixed with 4% paraformaldehyde, the cells were subjected to immunostaining as described above. The number of three-way junctions was counted in the same manner as described above to quantify the effects of Arl6IP5 knockdown, Arl6IP1 knockdown, and exogenous expression of the HA-tagged proteins following knockdown on the ER morphology.

For analysis of the ER-phagy flux, the cells were transfected with mCherry-GFP-FAM134B 3 days after transfection with the *Arl6IP5* siRNAs and cultured for only 6 h to avoid overexpression of mCherry-GFP-FAM134B. After being fixed with 4% paraformaldehyde, the cells were directly viewed without permeabilization using the confocal imaging system (ZEISS, LSM700) or a fluorescent microscope (ZEISS, Axio Lab. A1). Cells expressing high levels of mCherry-GFP-FAM134B were excluded from analysis, because overexpression of mCherry-GFP-FAM134B often impaired the overall morphology of the ER.

### Immunoprecipitation

Appropriate combinations of the plasmids were transfected into HEK293 cells using PEI. The day after transfection, the cells were harvested and disrupted in buffer A (20 mM Tris–HCl pH 7.5, 150 mM NaCl, 0.5 mM MgCl_2_) by sonication. The samples were solubilized with 0.5% Triton X-100 for 30 min, followed by centrifugation at 11,400*g* for 30 min. The Triton X-100 extracts were then incubated with the mouse anti-FLAG mAb, followed by immunoprecipitation with protein G sepharose (GE healthcare). The samples were subjected to SDS-PAGE followed by immunoblotting with the rabbit anti-FLAG pAb and the rat anti-HA mAb.

For immunoprecipitation of endogenous FAM134B, HEK293 cells were solubilized with TritonX-100 in the same manner as described above except that buffer B (20 mM Tris–HCl pH 7.5, 150 mM NaCl) was used instead of buffer A. The Triton X-100 extracts were then incubated with the rabbit anti-FAM134B pAb, followed by immunoprecipitation. The samples were subjected to SDS-PAGE followed by immunoblotting with the rabbit anti-FAM134B pAb and the rabbit anti-Arl6IP5 pAb. The blots were developed by chemiluminescence (Nacalai Tesque, Chemi-Lumi One Super) and the blot images were captured by LAS-4000 (Cytiva).

### Quantitative RT-PCR

Total RNAs were isolated from U2OS cells transfected with the siRNAs using the Qiagen RNeasy Mini kit and reverse transcribed with SuperScript VILO (Invitrogen) into the cDNAs. Quantitative PCR was performed on LightCycler 480 Real Time PCR System (Roche) using KAPA SYBR FAST Master Mix (KAPA BIOSYSTEMS). The quantities of *Arl6IP5* and *Arl6IP1* transcripts were normalized to *GAPDH*. The primer pairs used are human *Arl6IP5* (forward, 5′-ctgctctattaccagaccaac-3′; reverse, 5′-ggctgcccacacaaaccctgtg-3′), human *Arl6IP1* (forward, 5′-agataatcgcagcaccaac-3′; reverse, 5′-cagaaacaccaaagaaaccac-3′), and human *GAPDH* (forward, 5′-agggctgcttttaactctggt-3′; reverse, 5′-ccccacttgattttggaggga-3′).

## Data availability

All data were contained within the article.

## Supporting information

This article contains supporting information.

## Conflicts of interest

The authors declare that they have no conflicts of interest with the contents of this article.
