# Supplementary material for: ADP ribosylation factor–like GTPase 6–interacting protein 5 (Arl6IP5) is an ER membrane-shaping protein that modulates ER-phagy
Source: J Biol Chem. 2025 Apr 8;301(5):108493. doi: 10.1016/j.jbc.2025.108493 (PMC12136792; doi:10.1016/j.jbc.2025.108493)
Supplement: Figure S1 [file mmc1.pdf]

Figure S1. Overexpression of Arl6IP1 induces the extensive network of the peripheral ER tubules

(**A**) Localization of Arl6IP1 at the ER. HeLa cells were transfected with HA-Arl6IP1, followed by immunostaining with the anti-HA mAb and the anti-PDI mAb. The images of the cell expressing HA-Arl6IP1 at the low level are shown. The upper panels are the images taken with the same setting of the confocal microscope (such as laser power, and gain and offset parameters) as those of the cells expressing HA-Arl6IP1 at the high level as shown in (**B**). The middle panels are the images taken by adjusting the microscope settings to detect ER localization of HA-Arl6IP1. Bars: 10 µm. The boxed area is enlarged and shown in the bottom panels. Bar: 5 µm. The rightmost graph in the middle panels shows fluorescence intensity profiles for HA-Arl6IP1 and PDI along the white arrow. The rightmost graph in the bottom panels shows the cytofluorogram and Rcoloc indicates Pearson's correlation coefficient. The images and results shown are representative of three independent experiments.

(**B**) Induction of the extensive network of the peripheral ER tubules and exclusion of PDI from the peripheral ER tubules by overexpression of Arl6IP1. The images of the cell expressing HA-Arl6IP5 at the high level are shown. The images are taken with the same setting of the confocal microscope as the images in the upper panels in (**A**), highlighting the difference in extent of expression of HA-Arl6IP1. Bar: 10 µm. The boxed area is enlarged and shown in the bottom panels. Bar: 5 µm. The rightmost graph in the upper panels shows fluorescence intensity profiles for HA-Arl6IP1 and PDI along the white arrow. The images and results shown are representative of three independent experiments.

(**C**) Ratio of the cells expressing HA-Arl6IP1 at the high level. Sixty transfected cells were randomly chosen and the number of the cells expressing HA-Arl6IP1 at the high level was counted. The mean value and standard deviation of three independent experiments is shown. Each dot represents the result of a single experiment.

(**D**) Ratio of the cells having the extensive network of ER tubules induced by overexpression of HA-Arl6IP1. Thirty cells expressing HA-Arl6IP1 at the high level were randomly chosen and the number of the cells having the extensive network of ER tubules, as judged by the presence of unbranched, long ER tubules, was counted. The mean value and standard deviation of three independent experiments is shown. Each dot represents the result of a single experiment.
