# Supplementary figures and images for: ADP ribosylation factor–like GTPase 6–interacting protein 5 (Arl6IP5) is an ER membrane-shaping protein that modulates ER-phagy
Source: J Biol Chem. 2025 Apr 8;301(5):108493. doi: 10.1016/j.jbc.2025.108493 (PMC12136792; doi:10.1016/j.jbc.2025.108493)

# Figure S1

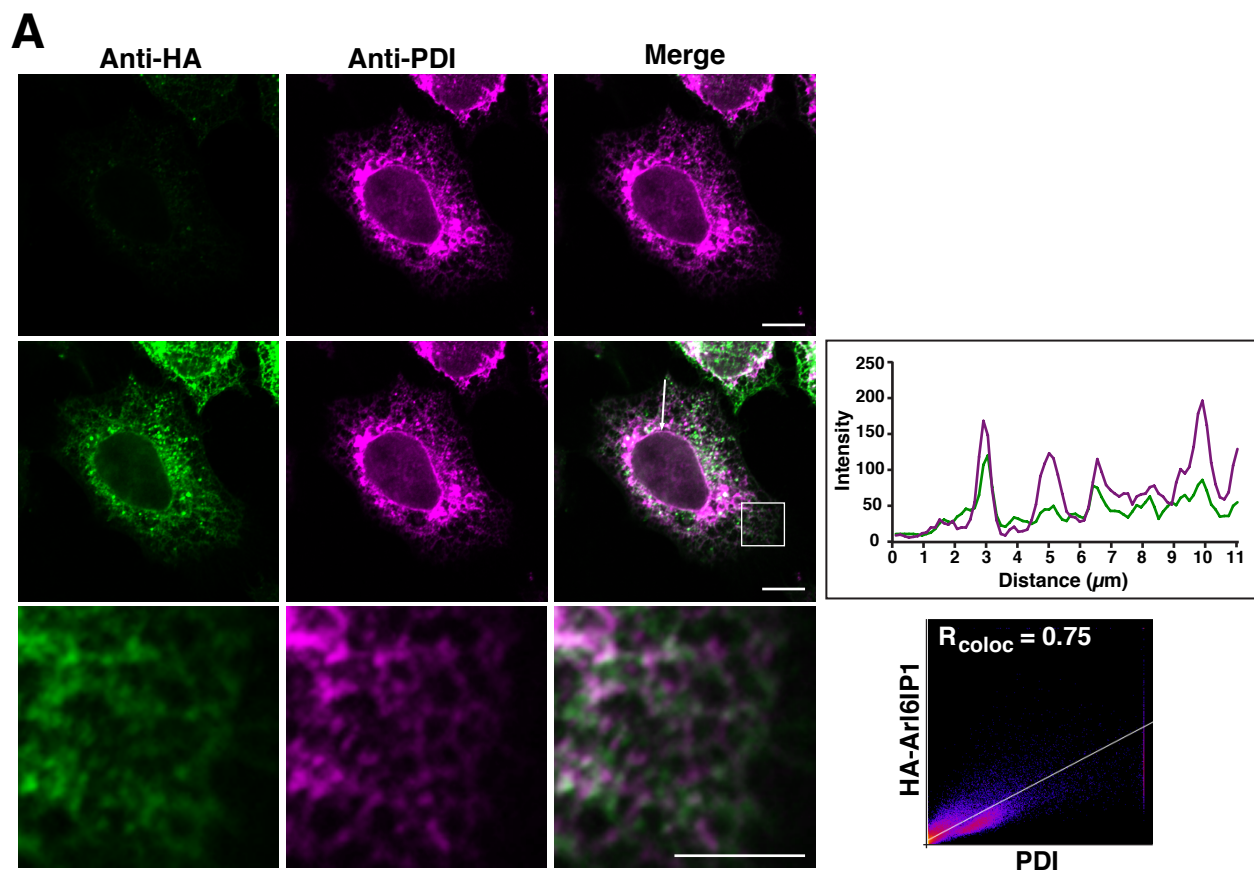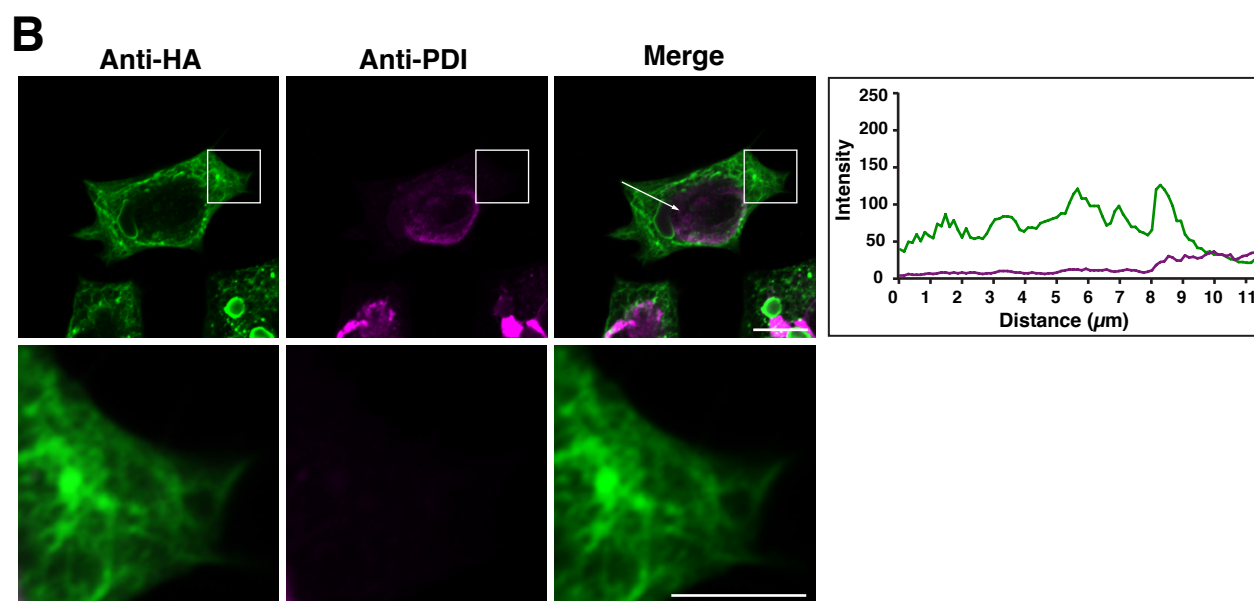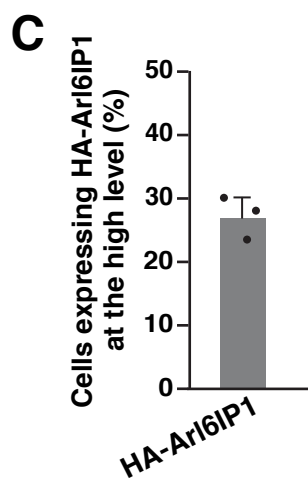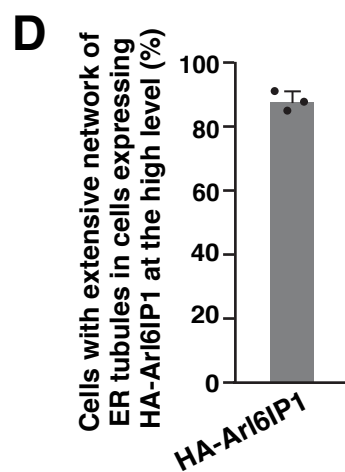

Supplement: Figure S2 [file mmc2.pdf]
