# Supplementary material for: ADP ribosylation factor–like GTPase 6–interacting protein 5 (Arl6IP5) is an ER membrane-shaping protein that modulates ER-phagy
Source: J Biol Chem. 2025 Apr 8;301(5):108493. doi: 10.1016/j.jbc.2025.108493 (PMC12136792; doi:10.1016/j.jbc.2025.108493)
Supplement: Figure S3 [file mmc3.pdf]

Figure S2

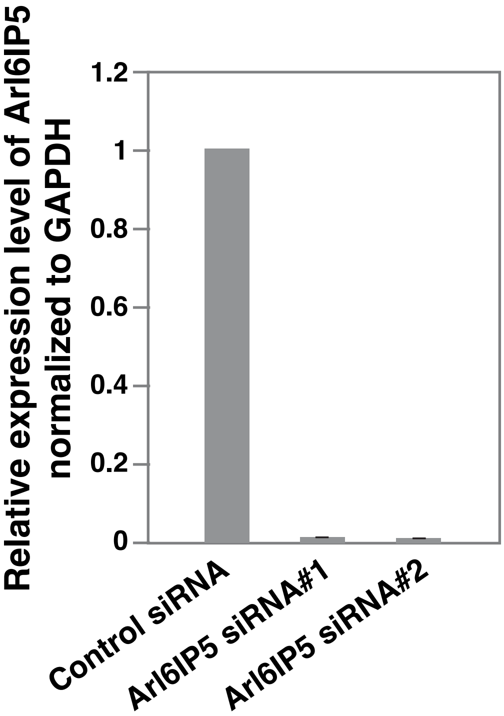

Figure S2. Validation of the siRNA-mediated knockdown of Arl6IP5 by quantitative RT-PCR
Total RNAs were isolated from U2OS cells transfected with the siRNAs targeting *Arl6IP5* or the control siRNA and reverse transcribed into the cDNAs, followed by quantitative RT-PCR. The quantities of *Arl6IP5* transcripts were shown relative to *GAPDH*. The error bars represent standard deviations of three independent measurements.
