# Supplementary material for: ADP ribosylation factor–like GTPase 6–interacting protein 5 (Arl6IP5) is an ER membrane-shaping protein that modulates ER-phagy
Source: J Biol Chem. 2025 Apr 8;301(5):108493. doi: 10.1016/j.jbc.2025.108493 (PMC12136792; doi:10.1016/j.jbc.2025.108493)
Supplement: Figure S5 [file mmc5.pdf]

# Figure S4

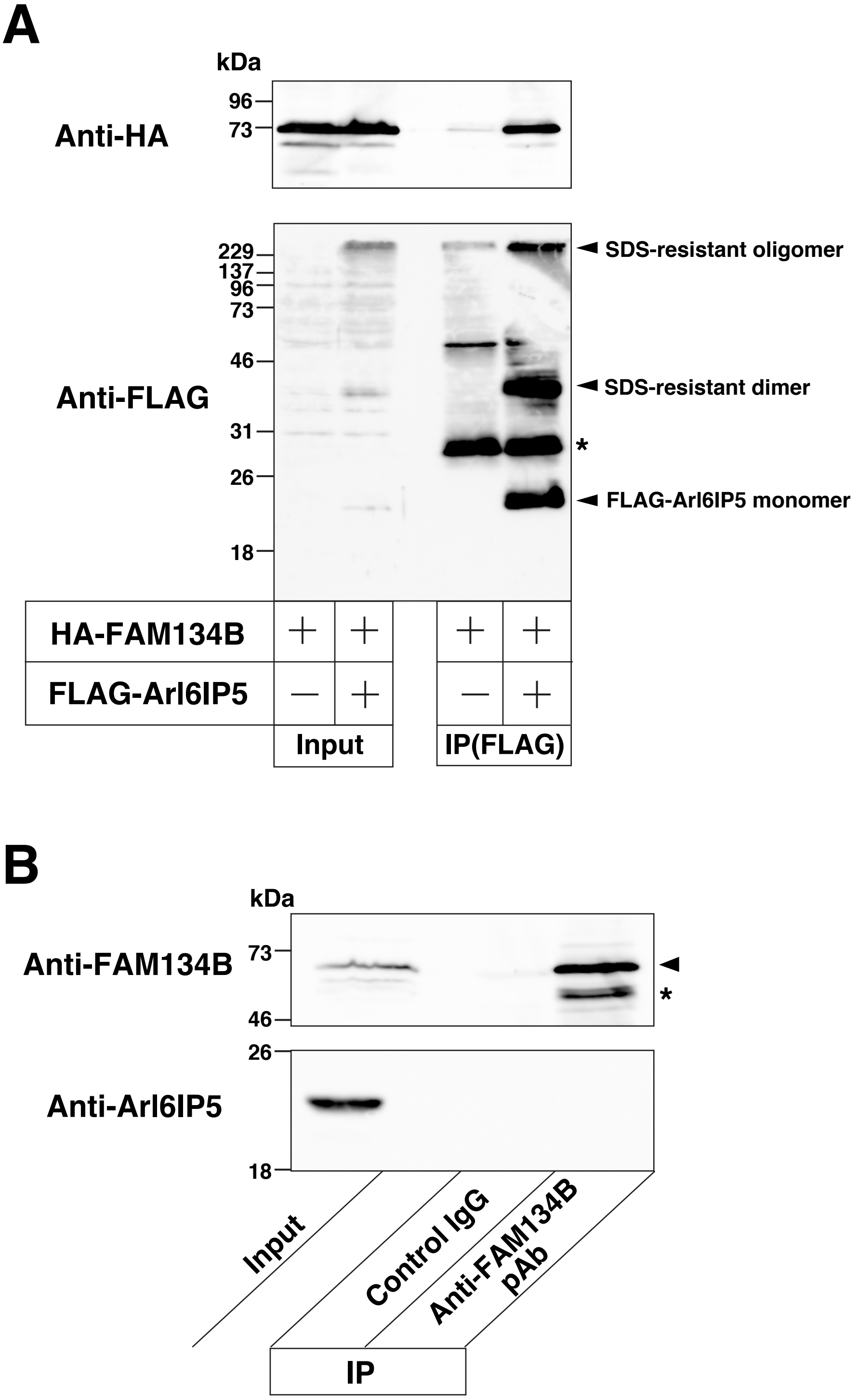


**Figure S4. Assessment of the interaction between Arl6IP5 and FAM134B**

(**A**) Coimmunoprecipitation of exogenous Arl6IP5 and exogenous FAM134B. The indicated combinations of FLAG-Arl6IP5 and HA-FAM134B were transfected into HEK293 cells, followed by immunoprecipitation with the anti-FLAG mAb. The samples were subjected to SDS-PAGE followed by immunoblotting with the anti-HA mAb and the anti-FLAG pAb. The asterisk indicates the non-specific bands from the light chain of IgG.

(**B**) Non-detection of coimmunoprecipitation of endogenous Arl6IP5 and endogenous FAM134B. HEK293 cells were solubilized with TritonX-100, followed by immunoprecipitation with the anti-FAM134B pAb. The samples were subjected to SDS-PAGE followed by immunoblotting with the anti-Arl6IP5 pAb and the anti-FAM134B pAb. The arrowhead indicates endogenous FAM134B (FAM134B-1). The band indicated by the asterisk is probably the short isoform of endogenous FAM134B (FAM134B-2) or degraded FAM134B.
